# Amplitude Integrated Electroencephalogram Study of the Effect of Caffeine Citrate on Brain Development in Low Weight Infants with Apnea

**Published:** 2019-07

**Authors:** Dong YANG, Bin ZHOU, Bao JIN, Xiao LIU, Yun WANG

**Affiliations:** Department of Paediatrics, Xuzhou Central Hospital, Xuzhou 221000, P.R. China

**Keywords:** Apnea and low birth weight, Citrate caffeine, Aminophylline, Brain development, Amplitude integrated EEG

## Abstract

**Background:**

To investigate the effect of caffeine citrate on the integrated brain electroencephalogram (EEG) of apnea and low birth weight infants.

**Methods:**

Overall, 212 infants with apnea and low birth weight admitted to Xuzhou Central Hospital from June 2016 to June 2018 were enrolled. The infants were divided into control group and observation group according to the random number table method, 106 cases in each group. Infants in control group were treated with aminophylline, and infants in the observation group were given caffeine citrate. All children were continuously tested by digital amplitude integrated brain function monitor. The amplitude-integrated electroencephalogram (aEEG) was used to detect sleep arousal cycle (Cy), graphic continuity (Co), lower edge amplitude value (LB) scores, aEEG continuous voltage and periodic occurrence rate, narrowband voltage and bandwidth.

**Results:**

After treatment, scores of Cy, Co and LB increased in both groups, and the scores were significantly higher in observation group than in control group (*P*=0.029, 0.017, 0.047). After treatment, continuous voltage positive rate, sleep-wake cycle occurrence rate, and narrow-band lower boundary voltage increased in both groups, and the values were significantly higher in observation group than in control group (*P*=0.011, 0.042). After treatment, aEEG detection bandwidth and the upper boundary voltage of the narrow band decreased in both groups, and the values were significantly lower in observation group than in control group (*P*=0.007, 0.020, 0.032).

**Conclusion:**

Citrate caffeine can alleviate the brain development of low-weight infants with apnea, improve brain electrical activity and promote brain function and maturity.

## Introduction

Apnea refers to a breathing stoppage time of more than 20s, or less than 20s but accompanied by bun or/and slow heart rate, which may cause reflex bradycardia, hypoxemia, etc. Some infants with apnea require mechanical ventilation. If there is no timely and effective treatment, continuous apnea will damage the developing brain (1). In recent years, with the continuous development of medical technology, survival rate of ultra-low and very low birth weight infants has increased significantly. Therefore, white matter damage now is the most common form of brain injury in premature infants, and its incidence is increasing year-by-year (2).

Amplitude-integrated EEG is a simple EEG monitoring. It is easy to operate, able to achieve continuous detection, intuitive and convenient, non-invasive, can effectively help to determine the brain development of premature infants, and has satisfactory predictive value for neurological damage and prognosis in infants (3). Aminophylline is often used in the treatment of children with apnea in the clinic. By promoting the sensitivity of the respiratory system to carbon dioxide, aminophylline can increase the respiratory rate and reduce the incidence of apnea, but its therapeutic concentration is similar to IC50, which often causes various adverse reactions (4). Caffeine citrate is a methylxanthine, which has a long half-life and has less toxic side effects. It is not necessary to monitor blood concentration of caffeine citrate in clinical practice, so it is considered a promising drug in the treatment of apnea and low birth weight infants (5).

Therefore, we investigated the effect of caffeine citrate on the integrated brain EEG of apnea and low birth weight infants.

## Methods

### General Information

Overall, 212 infants with apnea and low birth weight admitted to Xuzhou Central Hospital from June 2016 to June 2018 were enrolled in the study. According to random number table method, they were divided into control group and observation group, 106 cases in each group. There were 69 males and 37 females in observation group. The mean gestational age was (32.56 ± 2.35) weeks and the mean birth weight was (1.88 ± 0.42) kg. Sixty-five cases were nature labor infants and 41 cases were cesarean section infants. In control group, there were 63 males and 43 females. The mean gestational age was (33.02 ± 1.98) weeks and the mean birth weight was (1.93 ± 0.56) kg. Sixty-nine cases were nature labor infants and 37 cases were cesarean section infants. There were no significant differences in general data between the two groups.

Inclusion criteria: clinically diagnosed as low-weight children with apnea; younger than 12 h; children with body mass not exceeding 2500 g; parents who volunteered to participate in this study and have signed informed consent.

Exclusion criteria: primary lung disease or severe infection; infants with congenital dysplasia and with one or more congenital abnormalities that endanger life; infants with intracranial hemorrhage; infants with congenital chromosomal abnormalities or malformations; infants with obstructive apnea.

The study was approved by the Ethics Committee of Xuzhou Central Hospital.

### Methods

Infants in control group were treated with aminophylline (Ruiyang Pharmaceutical Co., Ltd.; State approval number: H20050415). The first dose was 5 mg/kg through intravenous pumping, and the maintenance dose was 2 mg/kg after 12 h, 12 h/time. Infants in observation group were given caffeine citrate (Sinopharm Group Co. Ltd State approval number: H20183216). The first dose was 20 mg/kg through intravenous pumping, and the maintenance dose was 5 mg/kg after 24 h, 24 h/time.

### Observation indicators

aEEG scores Cy, Co, LB, continuous voltage and cycle occurrence, narrowband voltage and bandwidth were compared between two groups. 1) Cy: no period is 0 points; first waveform is 1 point; an ambiguous period is 2 points; a clear period with interruption is 3 points; a clear period without interruption is 4 points; a maturity period of more than 20 minutes is 5 points. 2) Co score: pathological type is 0 points; discontinuous low voltage type lower bound amplitude <3 μV and upper boundary between 15 μV and 30 μV is 1 point; discontinuous high voltage type lower bound amplitude between 3 μV and 5 μV and upper boundary between 20 μV-40 μV is 2 points; continuous lower bound amplitude >5 μV and the upper boundary between 20 μV and 40 μV is 3 points. 3) LB score: <3 μV severe inhibition is 0 points; 3 μV-5 μV partial inhibition is 1 point; >5 μV no inhibition is 2 points. 4) Voltage: Discontinuity voltage indicates aEEG maximum amplitude >10 μV, minimum amplitude <5 μV; continuous voltage indicates the lowest amplitude is 7 μV-10 μV; partial amplitude reaches 10 μV-25 μV; the highest amplitude is 10 μV-25 μV. 5) Periodicity: amplitude and continuity activity last for more than 20 min; broadband is discontinuous EEG background activity; narrow band is continuous EEG background activity, i.e. no periodicity, immature period or maturity cycle. 6) Bandwidth: <15 μV for low bandwidth, 15 μV-20 μV for medium bandwidth, and >20 μV for high bandwidth.

### Statistical analysis

Statistical analysis was performed using SPSS 20.0 software package (Chicago, IL, USA). *t* test and x2 test were used to compare measurement data the count data, respectively. Difference was statistically significant at *P*<0.05.

## Results

### Comparison of Cy, Co, and LB scores detected by aEEG

There was no significant difference in scores of Cy, Co and LB between observation group and control group before treatment (*P*=0.078, 0.090, 0.055). After treatment, scores of Cy, Co and LB detected by aEEG increased in both groups, and the scores were significantly higher in observation group than in control group (*P*=0.029, 0.017, 0.047) (Table 1).

**Table 1:** Comparison of Cy, Co, and LB scores detected by aEEG

***Groups***	***Cases (n)***	***Cy score***		***Co score***		***LB score***	
		***Before treatment***	***After treatment***	***Before treatment***	***After treatment***	***Before treatment***	***After treatment***
Observation	106	1.80±1.21	2.48±1.07	2.22±1.07	2.85±1.55	1.81±1.20	2.67±0.83
Control	106	1.78±1.30	2.11±1.23	2.25±1.14	2.51±1.43	1.83±1.17	2.21±1.12
*t*		1.806	2.435	1.683	2.537	1.949	1.996
*P*		0.078	0.029	0.090	0.017	0.055	0.047

### Comparison of aEEG continuous voltage and cycle occurrence rate

There was no significant difference in positive voltage positive rate and sleep-wake cycle between observation group and control group before treatment (*P*=0.081, 0.057). After treatment, continuous voltage positive rate, sleep-wake cycle occurrence rate, and narrow-band lower boundary voltage increased in both groups, and the values were significantly higher in observation group than in control group (*P*=0.011, 0.042) (Table 2).

**Table 2:** Comparison of aEEG continuous voltage and cycle occurrence rate

***Groups***	***Cases (n))***	***Continuous voltage positive rate***		***Sleep-wake cycle occurrence rate,***	
		***Before treatment***	***After treatment***	***Before treatment***	***After treatment***
Observation	106	42 (39.62)	87 (82.07)	14 (13.20)	61 (57.54)
Control	106	45 (42.45)	69 (65.09)	12 (11.32)	34 (32.07)
*t*		1.653	8.036	3.158	4.739
*P*		0.081	0.011	0.057	0.042

### Comparison of aEEG narrow-band voltage and bandwidth

There was no significant difference in aEEG detection bandwidth, narrow-band upper boundary voltage and lower boundary voltage between observation group and control group before treatment (*P*=0.091, 0.059, 0.099). After treatment, aEEG detection lower boundary voltage of the narrow band increased in both two groups, and the values were significantly higher in observation group than in control group (*P*=0.007). After treatment, aEEG detection bandwidth and the upper boundary voltage of the narrow band decreased in both two groups, and the values were significantly lower in observation group than in control group (*P*=0.020, 0.032) (Table 3).

**Table 3:** Comparison of aEEG narrow-band voltage and bandwidth

***Groups***	***Cases (n)***	***Narrowband upper boundary voltage***		***Narrowband lower boundary voltage***		***Bandwidth***	
		***Before treatment***	***After treatment***	***Before treatment***	***After treatment***	***Before treatment***	***After treatment***
Observation	106	33.29±5.93	16.14±1.37	4.22±1.07	9.43±2.67	31.49±3.42	6.71±1.17
Control	106	31.08±5.21	22.33±2.14	4.35±1.16	7.74±2.01	30.08±3.15	17.04±1.98
*t*		1.679	2.793	1.939	2.511	1.649	2.389
*P*		0.091	0.007	0.059	0.020	0.099	0.032

## Discussion

Because of the immature development of the nerve center and respiratory center of premature infants, multiple apneas may occur, and the incidence is closely related to maturity. It has been reported (6) that the incidence of apnea in newborns with a birth weight of less than 1000 g is about 84%, the incidence of apnea in newborns born at 26 weeks to 27 weeks is about 78%; the incidence of apnea in neonates born at 30 weeks to 32 weeks is about 50%; at the incidence of apnea in neonates born at 34 weeks to 35 weeks is less than 10%. Repeated episodes of apnea can cause bradycardia, hypoxemia, etc., which causes brain damage in children and is serious life threatening. Methyl steroids are often used in the treatment of infants with apnea in clinic. The commonly used drugs are aminophylline and caffeine. Caffeine has a long half-life, and administered orally or intravenously. It can be administered once every 24 hours. In recent years, caffeine citrate has gradually become the drug of choice for the treatment of low-weight apnea patients (7). In this study, we investigated the effects of caffeine citrate on brain development in low-weight apnea infants by monitoring aEEG in infants.

The mechanism of action of aminophylline is to increase the sensitivity of central chemoreceptors to CO2, inhibit the degradation of intracellular cyclic adenosine monophosphate, increase the accumulation of intracellular cyclic adenosine monophosphate, stimulate the respiratory center to stimulate, promote diaphragm contraction, and increase cardiac output, and thus improve oxygenation status (8). However, due to the narrow range of the effective blood concentration of aminophylline, clinical dose cannot be well controlled. Low doses result in poor clinical efficacy, while high doses lead to tachycardia, nausea, irritability, feeding intolerance, fear of eating, increased blood sugar, impaired neutrophil function, reduced cerebral blood flow and other adverse reactions (9). The mechanism of actions of caffeine citrate and aminophylline are similar, citric acid can improve respiratory muscle function, and the respiratory excitatory effect is stronger. The effective blood drug concentration of caffeine citrate is wide and the half-life cycle is long, and it can be administered once every 24 hours, so the incidence of adverse reactions is low (10). aEEG is a simplified form of the original EEG. aEEG processes the brainwave activity signal by amplifying, filtering, and integrating the compressed amplitude, and traces it on the heat sensitive paper, so that the brain development of premature infants can be visualized (11). Therefore, this study comprehensively analyzed the periodicity, continuity, boundary amplitude, and spectral band bandwidth of aEEG brain wave background activity. Results showed that, after treatment, scores of Cy, Co and LB detected by aEEG increased in both groups, and the scores were significantly higher in observation group than in control group. After treatment, continuous voltage positive rate, sleep-wake cycle occurrence rate, and narrow-band lower boundary voltage increased in both groups, and the values were significantly higher in observation group than in control group (*P*<0.05). After treatment, aEEG detection bandwidth and the upper boundary voltage of the narrow band decreased in both two groups, and the values were significantly lower in observation group than in control group. These results suggest that the brain developmental maturity of the observation group is relatively better, which may be because high expression of adenosine can promote apoptotic activity, increase the level of apoptosis protease and free radicals, and then induce the potential inhibition. Therefore, blocking adenosine receptor expression in neonatal brain tissue can inhibit apoptosis (12). Caffeine citrate is a non-selective adenosine receptor inhibitor that increases the content of rough endoplasmic reticulum and free ribosome in hippocampal neurons, thereby regulating the expression of brain-derived neurotrophic factors and other related proteins. The development of neurological development improves the development of brain development (13).

## Conclusion

Caffeine citrate is more effective than aminophylline in relieving brain development in low weight apnea infants, and in improving brain electrical activity and promoting brain function maturation.

## Ethical considerations

Ethical issues (Including plagiarism, informed consent, misconduct, data fabrication and/or falsification, double publication and/or submission, redundancy, etc.) have been completely observed by the authors.
